# Patterns of prevalence and contemporary clinical management strategies in complicated acute biliary calculous disease: an ESTES ‘snapshot audit’ of practice

**DOI:** 10.1007/s00068-020-01433-x

**Published:** 2020-07-07

**Authors:** Gary Alan Bass, Amy Gillis, Yang Cao, Shahin Mohseni, for the European Society for Trauma, Emergency Surgery (ESTES) Cohort Studies Group, A. Shamiyeh, L. Rosetti, G. Klimbacher, B. Klugsberger, P. Healy, C. Moriarty, C. Power, N. Knightly, A. D. K. Hill, D. C. Winter, M. E. Kelly, B. E. Creavin, É. J. Ryan, C. C. Duffy, M. Sugrue, M. H. Moore, L. Flanagan, J. Ryan, C. Keady, B. Fahey, K. L. McKevitt, K. Barry, K. C. Conlon, K. Mentor, A. Kazemi-Nava, B. J., P. F. Ridgway, D. O. Kavanagh, M. Whelan, M. Donnelly, C. McCarrick, U. Muhammad, T. M. Connelly, P. C. Neary, S. Magalina, V. Cozza, A. LaGreca, D. Gui, A. Malagnino, M. Zago, M. Montuori, A. Biloslavo, N. Samardzic, S. Fracon, D. Cosola, N. de Manzini, U. Fernandes, P. Avelar, R. Marques, A. S. Esteves, A. Marçal, C. Gomes, D. Machado, T. Teles, S. Neves, M. Semiao, R. Cunha, J. Pereira, J. Constantino, M. Sá, C. Casimiro, L. Ionescu, R. Livadariu, L. Stirbu, R. Danila, D. Timofte, B. Astefaniei, A. Landaluce Olavarria, B. Estraviz Mateos, J. Gonzalez Taranco, D. Gomez, J. Barrutia, J. Zeballos, D. Morales Garcia, A. Lozano Najera, E. Gonzalez Tolaretxipi, L. Tallon-Aguilar, J. Pintor-Tortolero, A. Sanchez-Arteaga, V. Duran-Muñóz Cruzado, V. Camacho-Marente, J. Tinoco-Gonzalez, A. Älverdal, S. Redeen, S. Mohseni, A. Mohammad, R. Ahl, M. Wikström, S. Marinos, N. Warner, R. Patel, T. Magro, R. Sunthareswaran, A. Mihailescu, G. Pokusewski, A. L. Bubuianu, C. Dimitriu, M. Paraoan, A. Desai, K. Jones, M. Mlotshwa, K. Ross, S. Lambracos, Y. Tryliskyy, D. C. Cullinane

**Affiliations:** 1 Emergency Surgery Committee, European Society for Trauma and Emergency Surgery (ESTES), Pölten, Austria; 2 Department of Surgery, Tallaght University Hospital, Dublin 24, Ireland; 3 Department of Surgery, Örebro University School of Medical Sciences, Örebro, Sweden; 4 Department of Traumatology, Surgical Critical Care and Emergency Surgery, University of Pennsylvania, Philadelphia, USA; 5 Department of Clinical Epidemiology and Biostatistics, School of Medical Sciences, Örebro University, Örebro, Sweden

**Keywords:** Cholecystitis, Clinical practice guidelines, Cholecystectomy, Emergency surgery

## Abstract

**Background:**

Acute complications of biliary calculi are common, morbid, and complex to manage. Variability exists in the techniques utilized to treat these conditions at an individual surgeon and unit level.

**Aim:**

To identify, through an international prospective nonrandomized cohort study, the epidemiology and areas of practice variability in management of acute complicated calculous biliary disease (ACCBD) and to correlate them against reported outcomes.

**Methods:**

A preplanned analysis of the European Society of Trauma and Emergency Surgery (ESTES) 2018 Complicated Biliary Calculous Disease audit was performed. Patients undergoing emergency hospital admission with ACCBD between 1 October 2018 and 31 October 2018 were included. All eligible patients with acute complicated biliary calculous disease were recorded contemporaneously using a standardized predetermined protocol and a secure online database and followed-up through to 60 days from their admission.

**Endpoints:**

A two-stage data collection strategy collecting patient demographics, details of operative, endoscopic and radiologic intervention, and outcome metrics. Outcome measures included mortality, surgical morbidity, ICU stay, timing of operative intervention, and length of hospital stay.

**Results:**

Three hundred thirty-eight patients were included, with a mean age of 65 years and 54% were female. Diagnosis at admission were: cholecystitis (45.6%), biliary pancreatitis (21%), choledocholithiasis with and without cholangitis (13.9% and 18%). Index admission cholecystectomy was performed in just 50% of cases, and 28% had an ERCP performed. Morbidity and mortality were low.

**Conclusion:**

This first ESTES snapshot audit, a purely descriptive collaborative study, gives rich ‘real world’ insights into local variability in surgical practice as compared to international guidelines, and how this may impact upon outcomes. These granular data will serve to improve overall patient care as well as being hypothesis generating and inform areas needing future prospective study.

## Introduction

Biliary calculi, while predominantly a common benign asymptomatic entity, may also produce acute complications—such as complicated cholecystitis, choledocholithiasis with/without cholangitis, and biliary pancreatitis—requiring urgent hospital presentation for surgical care [1, 2]. These complications may be morbid and are complex to manage [3–5]. Despite the frequency of presentation of these patients, there remains some clinical equipoise around the optimal timing of diagnostic investigations, the timing of surgical, endoscopic, or percutaneous interventional radiologic therapies [6–9].

For surgeon researchers wishing to investigate the heterogenous reality of daily surgical practice rather than the dichotomous outcome of RCTs, there formerly were few options other than retrospective analysis of large aggregated billing or census datasets. Although the development of comprehensive datasets (e.g., cancer registries from Dutch, Scandinavian, and UK investigators as well as the National Cancer Institute's SEER and the American College of Surgeons NSQIP databases) improved the granularity and appropriateness of the source data to disease-specific interrogation, they still suffered the limitations of retrospective analyses, and some have questioned the broad applicability of conclusions drawn [10]. Since 2015, however, the European Society of Coloproctology (ESCP) and others pioneered the ‘snapshot audit’—a novel methodology for prospective collaborative observational cohort studies that has allowed detailed, defined datasets to be accrued in line with a priori analyses stated in prepublication, open access protocols filed with clinical trial repositories [11–13].

The European Society for Trauma and Emergency Surgery (ESTES), seeking to evaluate real world experience with the contemporary management of complicated biliary calculous disease, have adopted the ‘snapshot audit’ methodology in the current study (ClinicalTrials.gov Trial #NCT03610308) in order to better define the epidemiology, management, and outcomes in patients with ACCBD among ESTES participating centers.

We present a preplanned analysis of the European Society of Trauma and Emergency Surgery (ESTES) 2018 Complicated Biliary Calculous Disease ‘Snapshot Audit’ was performed. This 30-day prospective cohort study, coordinated by representatives of the Emergency Surgery Committee of European Society of Trauma and Emergency Surgery, was performed across Europe in late autumn/winter 2018. Conscious of the challenges and limitations of this type of study, we however endeavored to capture unvarnished differences in epidemiology and clinical practice patterns across our contributing centers in order to inform future study.

## Methods

### Protocol

A prospective, observational, nonrandomized multicenter cohort study was conducted in line with a prespecified protocol which was registered with ClinicalTrials.gov (Trial # NCT03610308). The study enrolled all consecutive patients admitted with complicated biliary calculous disease during the month of October 2018 and followed those patients for 60 days post admission (up to December 31st, 2018). The database was closed for analysis on February 1st, 2019. In May 2019, an anonymized follow-up survey was completed by all 25 centers, assessing self-reported awareness of and adherence to recommendations outlined in the expert consensus Tokyo Guidelines (TG18), last updated in 2018 [14].

### Center eligibility

Any unit undertaking caring for emergency general surgical patients was eligible to register to enter patients into the study. No minimum case volume, or center-specific limitations were applied. Centers were asked to classify the model of unscheduled surgical care employed at their surgical department/hospital into one of the following categories: a dedicated Acute Care/Emergency Surgery service line (separate from elective surgical care), vs*.* a traditional ‘on call’ emergency service provided by general surgeons (e.g., upper GI, breast, hepatobiliary, or colorectal) with a primary commitment to elective surgical care. The study protocol was disseminated to registered members of the European Society of Trauma and Emergency Surgery (ESTES), and through national surgical societies.

### Patient eligibility

**Fig. 1:**
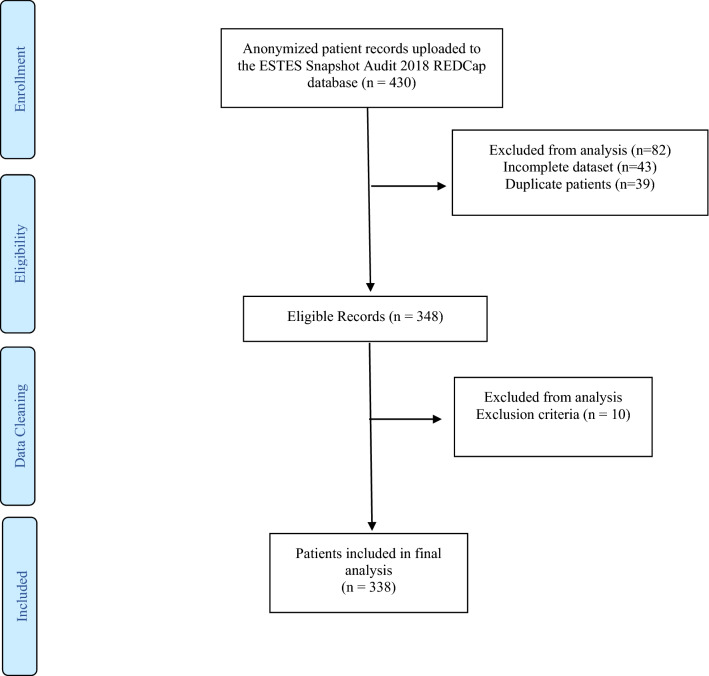
Patient flow diagram

All adult patients (over 18 years of age) admitted for acute gangrenous or perforated calculous cholecystitis (American Association for the Surgery of Trauma AAST Severity Grade II or above), choledocholithiasis or complications of cholelithiasis, and/or choledocholithiasis, or biliary pancreatitis were included in the current study [15, 16]. Patients presenting with biliary colic or Sphincter of Oddi dysfunction during the studied period were not eligible for inclusion (Fig. 1). Surgical procedures performed on these patients included cholecystectomy (open, laparoscopic, or laparoscopic converted to open), choledochotomy/common bile duct exploration (open or laparoscopic) or pancreatic necrosectomy. The data on endoscopic retrograde choledochopancreatography (ERCP), radiologic percutaneous cholecystostomy (transhepatic or transperitoneal), percutaneous transhepatic drainage, stone removal, or stent placement were also collected. Patients with uncomplicated biliary colic or biliary dyskinesia were excluded from the study.

### Data capture

The data were recorded contemporaneously and stored on a secure, user-encrypted online platform (REDCap^®^) without patient-identifiable information. Centers were obliged to validate that all eligible patients during the study period had been entered, and to attain > 95% completeness of data field entry prior to final submission. Quality assurance mentorship was provided by at least one consultant/attending-level surgeon at every participating site.

### Outcome measure

The primary outcome measure was index admission surgical definitive treatment. The secondary outcome measures were length of stay, the postoperative major complication rate defined as Clavien–Dindo classification [17] grades 3–5 (reoperation, reintervention, unplanned admission to intensive care unit, organ support requirement, or death), the postoperative length of stay (in whole days), with day of surgery as day zero, and the postoperative mortality rate, defined as death within 30 days of surgery.

### Statistical analysis

The descriptive and inferential statistical analyses were performed using the Jamovi project version 1.2.22.0 (www.jamovi.com, 2020) utilizing the R language for statistical computing. Effect estimates are presented as odds ratios (OR) with 95% confidence intervals and two-tailed *P* values. An alpha significance level of 0.05 was used through-out. Measures of central tendency were presented as mean ± standard deviation (range), or as median (interquartile range), as appropriate.

### Ethical considerations

All participating centers had institutional review board approval or equivalent. No patient consent was sought since the current study was purely observational and did not change the medical course of any patient. All data were de-identified at source when uploaded to the secure study database.

## Results

### Participating centers

**Fig. 2:**
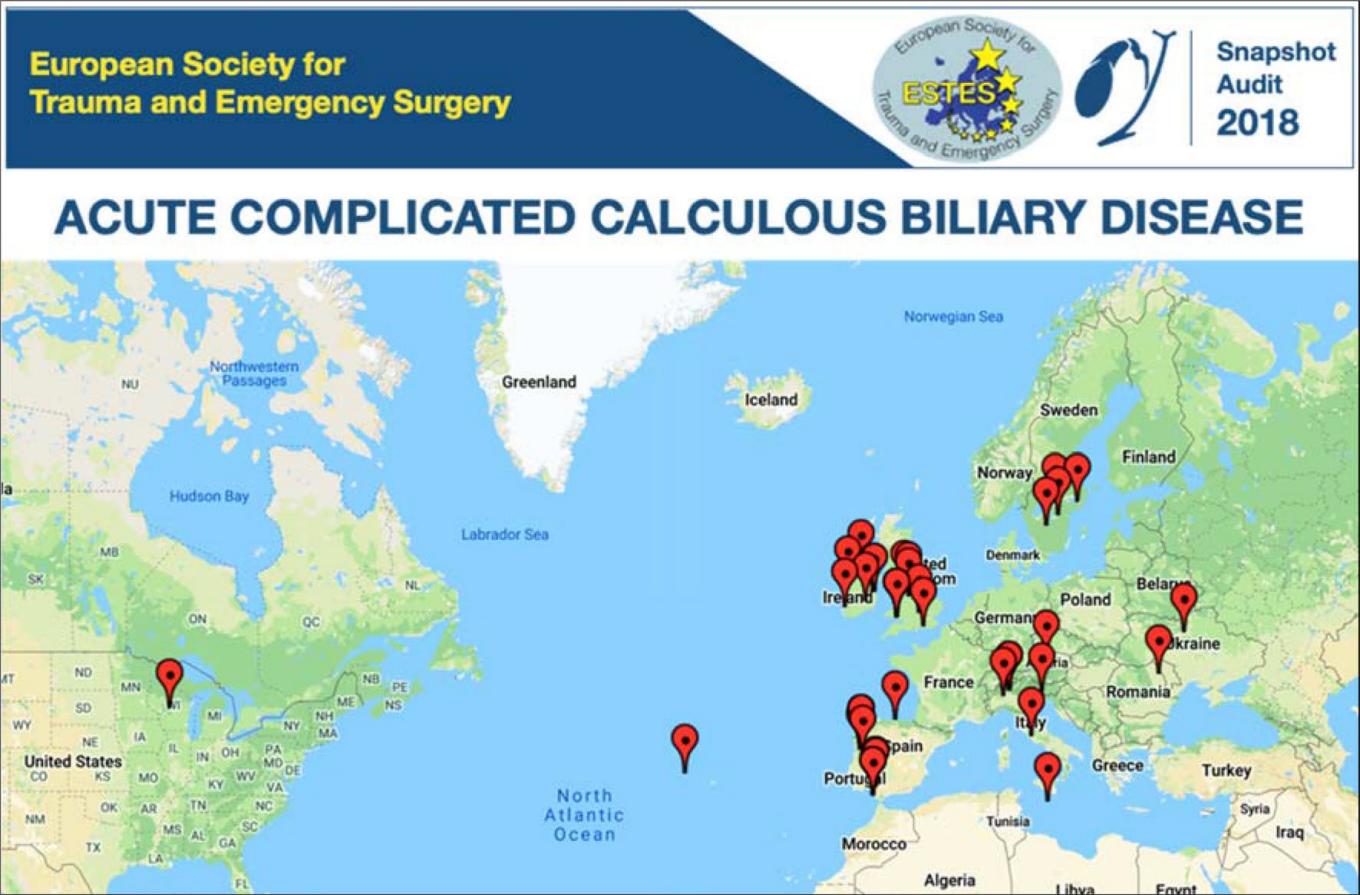
Twenty-five centers in nine countries (Austria, Ireland, Italy, Portugal, Romania, Spain, Sweden, UK, USA) participated in the inaugural ESTES Snapshot Audit

After an open call for participation by ESTES in May 2018, 25 centers from 9 countries (Austria, Ireland, Italy, Portugal, Romania, Spain, Sweden, UK, USA) completed the local ethics approval process and proceeded to prospectively enroll patients (Fig. 2). Fourteen centers (54%) described themselves as a University Hospital/Tertiary Referral Center, while (46%) described themselves as a General/Community Hospital. The median catchment population of each center was 500,000 people. The majority of centers reported high volumes of appropriate cases, with 22 (88%) centers performing more than 100 elective laparoscopic cholecystectomies per year. Similarly, 24 (96%) centers reported receiving greater than 300 admissions per annum for symptomatic biliary calculous disease.

### Model of unscheduled surgical care

A dedicated Acute Care/Emergency Surgery (ACS) service line (separate from elective General Surgery) existed in 7 (28%) centers, while General Surgery ‘on call’ managed and operated on patients in 18 (72%) centers (Table 1). Prior training in HPB surgery was declared by surgeons in 8 (32%) responding centers (Table 1). No difference in declared prior HPB surgical training was noted between centers utilizing an ACS model of care vs the traditional general surgery ‘on call’ model (*p* = 0.893). HPB-trained surgeons operated on 67/140(48%) vs. 102/198 (51.5%) patients of the total cohort of performed cholecystectomies in the current study (*p* = 0.503). Postoperative complications were seen in 14 (13%) cases operated by non HPB-trained vs. 8 (11.9%) cases operated by HPB-trained surgeons (*p* = 0.185). HPB-trained surgeons experienced 4 out of 8 reported bile duct injuries; as well as one serious hemorrhage and one enterotomy, both requiring return to the operating room. ACS service lines took care of 37% patients in this study, while 63% were under the care of General Surgery services (via ‘on call’ cover). Cholecystectomy was performed in 60% (75/125) of ACS patients, with 94.6% (*n* = 71) performed during the index admission and 5.4% (*n* = 4) as a planned interval re-admission; while 44.1% (94/213) underwent cholecystectomy under the care of the General Surgery ‘on call’ service [86.2% (*n* = 81) during the index admission and 13.8% (*n* = 13) as a planned interval re-admission]; *p* = 0.149.

**Table 1:** Demographic characteristics of centers

Who provides unscheduled surgical care?	
General surgery on-call	18 (72%)
Acute care surgery	7 (28%)
Undertaken training in hepatobiliary surgery?	
No	17 (68%)
Yes	8 (32%)

### Patient demographics

All of the 338 consecutive patients enrolled in the study were followed up until 60 days postadmission (latest patient 31st December 2018), (Fig. 1). Female patients outnumbered male (53.8% vs. 46.2%). The median age at the time of diagnosis was 67 ± 18 years (range 18–100 years) (Table 2).

**Table 2:** Demographic characteristics of patients

	Total 338
Age, mean (SD)	64.5 (18.4)
Sex,* n* (%)	
Male	156 (46.2%)
Female	182 (53.8%)
BMI, mean (SD)	28.5 (6.4)
Smoking,* n* (%)	
Smoker	165 (48.8%)
Nonsmoker	55 (16.3%)
Ex-smoker > 6 weeks	118 (34.9%)
American Society of Anesthesiology Status,* n* (%)	
1	62 (18.6%)
2	149 (44.7%)
3	95 (28.5%)
4	27 (8.1%)
Age-adjusted Charlson co-morbidity index, mean (SD)	3.36 (2.6)
APACHE-II score, mean (SD)	12.3 (7.59)
Admitting diagnosis,* n* (%)	
Cholecystitis	154 (45.6%)
Biliary pancreatitis	71 (21.0%)
Choledocholithiasis with cholangitis	47 (13.9%)
Choledocholithiasis without cholangitis	61 (18.0%)
Bilio-enteric fistula	5 (1.5%)

The median body mass index (BMI) was calculated as 27.34 ± 6.14 kg/m^2^ (range 15.9–69.6 kg/m^2^). Patients who currently smoke tobacco products accounted for 16.3% of the cohort, while there were 34.9% ex-smokers (> 6 weeks prior to admission), and 48.8% patients who had never smoked. The median (IQR, mean ± SD) age-adjusted Charlson co-morbidity index was 3 (1–5; 3.36 ± 2.6) while the mean (SD) APACHE-II score was 12.3 (7.59) (Table 2).

### Diagnosis

Acute calculous cholecystitis was present in 45.6% (*n* = 154) of the cohort, acute biliary pancreatitis in 21% (*n* = 71), choledocholithiasis in 31.9% (*n* = 108) of whom 43.5% (*n* 47) had cholangitis. Five patients (1.5%) were admitted for treatment of Mirizzi syndrome or bilioenteric fistula (Table 2). Acute cholecystitis was graded using the American Association for the Surgery of Trauma (AAST) Emergency Surgery grading system. One hundred twenty-five patients (80.6%) were AAST grades I–II (complicated acute cholecystitis, gallbladder empyema, or gangrenous cholecystitis), 19 (12.3%) were AAST grade III (contained/sealed gallbladder perforation), 9 (5.8%) were AAST Grade IV (pericholecystic abscess) and 2 (1.3%) were AAST grade V (free perforation with peritonitis).

Acute biliary pancreatitis was also graded using the AAST Emergency Surgery grading system. Interstitial pancreatitis was seen in 67 (94.4%) of patients with pancreatitis—57 (80%) were AAST grade I and 10 (14.3%) were AAST grade II. Just 3 (4.3%) patients had necrotizing pancreatitis, 2 (2.8%) were AAST grade III and 1 patient (1.4%) was AAST grade IV.

### Clinical features

Right upper quadrant (RUQ) abdominal pain was present on index admission in 312 (92.3%), nausea and vomiting in 182 (53.8%), fever in 82 (24.3%), anorexia in 59 (17.5%), jaundice in 40 (11.8%), rigors in 19 (5.6%), and pruritus in 9 (2.7%) of patients. The classical Charcot’s triad of RUQ pain, fever, and jaundice was found in 59.6% (28/47) of cholangitic patients.

### Diagnostic radiologic investigations

#### Ultrasound

An abdominal ultrasound (US) was performed as diagnostic investigation in all 338 study patients; all patients were reported to have cholelithiasis. Sonographic gallbladder wall thickening was reported in 119 (35.2%) patients, pericholecystic fluid in 48 (14.2%) patients, while biliary ductal dilatation was noted in 88 (26%) and gallbladder perforation in 19 (5.6%).

#### Computed tomography (CT)

Abdominal computed tomography (CT) was performed in 159 (47%) of patients in the study. Gallbladder perforation was reported in 24 (15%), biliary ductal dilatation was seen in 74 (46.5%) patients, abscess or empyema was reported in 34 (21.4%) and biloma in 2 (1.2%) of the CTs performed. The Balthazar CT pancreatitis severity score was reported in just 37 (23%) patients: 24 as mild pancreatitis, 21 moderate pancreatitis, and 3 severe necrotizing pancreatitis.

#### Magnetic resonance cholangiopancreatography (MRCP)

Magnetic resonance cholangiopancreatography (MRCP) was performed in 162 (47.9%) of patients in the study. No pathology was reported in 77 (47.5%) of MRCPs performed. Biliary ductal dilatation was reported in 58 (35.8%), and choledocholithiasis was reported in 67 (41.4%), gallbladder perforation in 5 (3%), abscess, or gallbladder empyema in 5 (3%) and biloma in 1 (0.6%).

### Operative management

Of the 338 patients enrolled in the study, 169 (50%) underwent surgical intervention, while 169 (50%) had not received operative treatment by the end of the 60-day follow-up period. Of those subjected to operative management, 151 patients (89.9%) underwent surgical intervention during the index admission, while a further 17 (10.1%) patients were reported as having been operated upon after discharge from index admission but prior to the closure of the study database (Table 3).

**Table 3:** Surgical, endoscopic and interventional radiologic management

Surgical intervention	169/338 (50%)
During the index admission	152 (89.9%)
Cholecystectomy	152 (100%)
Laparoscopic	127 (83.6%)
Conversion to open	13 (8.5%)
Open	12 (7.9%)
Subtotal cholecystectomy	5/152 (3.3%)
Laparoscopic	4
Conversion to open	1
Interval elective re-admission	17 (10.1%)
Cholecystectomy	17 (100%)
Laparoscopic	17 (100%)
Did not receive operative treatment	169/338 (50%)
Bile Duct management	104/338 (30.8%)
Intraoperative ERCP	11/104 (10.6%)
ERCP as separate procedure	87/104 (83.6%)
Intraoperative CBD exploration (laparoscopic)	4/104 (3.8%)
Intraoperative CBD exploration (open)	2/104 (1.9%)
Interventional radiology	26/338 (7.7%)
Cholecystostomy	23/26 (88.5%)
Drainage of abscess or fluid collection	1/26 (3.8%)
Percutaneous transhepatic cholangiography (PTC) ± drain	2/26 (7.7%)

Cholecystectomy was performed in 151 (99.3%) cases; the sole other operation performed was pancreatic necrosectomy (including splenic flexure colonic resection) in 1 patient (0.7%). Laparoscopic cholecystectomy was completed in 83.4% (126) of cases, including 4 laparoscopic subtotal cholecystectomies. Conversion to open cholecystectomy occurred in 13 (8.6%) cases, including 1 conversion for subtotal cholecystectomy (0.6%) and 1 for hepaticojejunostomy (0.6%). A further 12 (7.9%) cholecystectomies were performed as open from the beginning of the procedure. All five subtotal cholecystectomies and the hepaticojejunostomy were performed for cholecystitis. Surgical management of common bile duct calculi was undertaken in six patients—four laparoscopic and two at open surgery.

The mean time to from admission to operation in index admission was 2.66 ± 3.59 days (0–19 days) for all patients. During the index admission, the mean time from admission to operation was significantly shorter in patients with acute cholecystitis (1.74 ± 3.16 days) than in patients with biliary pancreatitis (5.06 ± 4.17 days, *p* = 0.002) or choledocholithiasis complicated by cholangitis (6.67 ± 5.54 days, *p* = 0.005), but not when compared with patients who had uncomplicated choledocholithiasis (3.7 ± 2.64 days, *p* = 0.125). Interval cholecystectomy was recorded in 17 patients, with a median interval to cholecystectomy from index admission of 66 (43–71) days. Mean(± SD; median, IQR) total length of in-patient stay (irrespective of diagnosis and whether or not the patient was operated) did not differ between patients treated by model of unscheduled care delivery (ACS vs General Surgery), at 7.95 (± 5.74; 7, 4–9) days for ACS versus 10.00 (± 10.8; 7, 4–11) days for General Surgery (*p* = 0.403); however, postoperative length of hospital stay (comprising just those patients who underwent surgery during the index admission) was significantly shorter in patients treated by ACS at 5.07(± 5.50; 3, 2–6) days vs 7.67(± 9.27; 4.5, 3–8) days (*p* = 0.007).

### Postoperative complications

Postoperative complications were infrequent in this snapshot audit—numbering 22 (13%) in 169 operated patients (). 21/152 (13.8%) following index admission surgical intervention versus 1/16 (6.25%) following interval elective surgery, *p* = 0.813. Infectious complications (deep abscess in 7 patients and superficial skin infection in 4 patients) represented the greatest number of postoperative complications, followed by bile duct injuries (*n* = 9, 5.3%), Strasberg Grade A (injury to small ducts in continuity with the biliary system, with a leak in the duct of Luschka or the cystic duct) in seven patients, Strasberg grade D (lateral injury to the extrahepatic biliary ducts) in one patient and Strasberg grade E2 (stricture < 2 cm from the bifurcation of the right and left bile ducts) in one patient, one postoperative hemorrhage requiring reoperation, and one enterotomy (identified and treated at time of index operation) (Table 4).

**Table 4:** Postoperative complications, categorized by timing of operation (index admission vs. interval cholecystectomy) and by Model of Unscheduled Surgical Care (elective General Surgery providing on-call cover vs. Acute Care Surgery)

Model of unscheduled care	Postoperative complications	Interval		Total
		index admission	Interval	
General surgery on-call	Abscess	3 (100%)	0	3 (100%)
	Bile duct injury	5 (83.3%)	1 (16.7%)	6 (100%)
	A	3 (75%)	1 (25%)	4 (100%)
	D	1 (100%)	0	1 (100%)
	E2	1 (100%)	0	1 (100%)
	Hemorrhage	1 (100%)	0	1 (100%)
	Wound infection	4 (100%)	0	4 (100%)
	Enterotomy	1 (100%)	0	1 (100%)
	Total	14 (93.3%)	1 (6.7%)	15 (100%)
Acute care surgery	Abscess	3 (100%)	0	3 (100%)
		100.0%	0.0%	100.0%
	Bile duct injury	3 (100%)	0	3 (100%)
	A	3 (100%)	0	3 (100%)
	Haemorrhage	0	0	0
	Wound infection	0	0	0
	Enterotomy	0	0	0
	Total	6 (100%)	0	6 (100%)
Total	Abscess	6 (100%)	0	6 (100%)
	Bile duct injury	8 (88.9%)	1 (11.1%)	9 (100%)
	A	6 (85.7%)	1 (14.3%)	7 (100%)
	D	1 (100%)	0	1 (100%)
	E2	1 (100%)	0	1 (100%)
	Hemorrhage	1 (100%)	0	1 (100%)
	Wound infection	4 (100%)	0	4 (100%)
	Enterotomy	1 (100%)	0	1 (100%)
	Total	19 (95%)	1 (5%)	20 (100%)

### Endoscopic management

Endoscopic evaluation and management of the common bile duct was undertaken in 98 (29%) of patients. Of these, Endoscopic Retrograde Cholangiopancreatography (ERCP) with duct clearance and sphincterotomy was performed in 76 (77.6%), ERCP and stent placement in 19 (19.4%) and EUS alone, without therapeutic duct management, was performed in 3 (3.1%). Intraoperative rendezvous ERCP during laparoscopic cholecystectomy was performed in 11 (11.2%) patients, while the remaining 87 (88.8%) patients underwent ERCP as a stand-alone procedure (with or without subsequent laparoscopic cholecystectomy). Of those patients undergoing ERCP, 9 (9.2%) patients experienced complications—post-ERCP pancreatitis in 6 (6.1%), bleeding in 3 (3.1%); no procedure was complicated by perforation. Median (IQR, mean ± SD) time from admission to endoscopy was 5.0 (2–8; 7.55 ± 9.86) days (Table 5).

**Table 5:** Antimicrobial therapy, microbiology specimens and organisms identified

Antimicrobial Prescription	307/338 (90.8%)
Piperacillin/tazobactam	133 (39.3%)
Amoxicillin/clavulanate	65 (19.2%)
Meropenem	23 (6.8%)
Metronidazole	15 (4.4%)
Cephalosporin	14 (4.1%)
Ciprofloxacin	13 (3.8%)
Gentamycin	10 (3.0%)
Ciprofloxacin and metronidazole	11 (3.3%)
Cephalosporin and metronidazole	11 (3.3%)
Amoxicillin/clavulanate and gentamycin	6 (1.8%)
Amoxicillin/clavulanate and metronidazole	6 (1.8%)
Did not receive antimicrobial treatment	31/338 (9.2%)
Blood and bile cultures	154/338 (45.5%)
Blood cultures	
Yes	47 (30.5%)
No	107 (69.5%)
Bile cultures	
Yes	35 (22.7%)
No	119 (77.3%)
Organism identified on culture	40 blood, 52 bile
*E. coli*	11 blood, 20 bile
*Klebsiella* spp.	2 blood, 4 bile
*Staphylococcus* spp.	3 blood, 3 bile
*Streptococcus* spp.	0 blood, 4 bile
*Enterococcus* spp.	0 blood, 4 bile
*Pseudomonas* spp.	1 blood, 1 bile
Other organisms	2 blood, 6 bile
Polymicrobial	1 blood, 10 bile
No growth	61 blood, 69 bile

### Interventional radiologic management

Interventional radiologic management of the gallbladder or common bile duct was undertaken in 26 (7.7%) of patients. Cholecystostomy was performed in 23 (88.5%) of these cases, percutaneous radiologic drainage of a collection, or abscess was performed in one (3.8%) patient and percutaneous transhepatic cholangiography was performed in two (7.7%). No complication was recorded for patients undergoing interventional radiologic procedures. Median (IQR, mean ± SD) time from admission to interventional radiology procedure was 2.0 (0–7; 3.89 ± 9.19) days (Table 5). Drain placement was homogenous across participating countries and was not affected by HPB availability or prior HPB training at an individual surgeon or center level (*p* = 0.360). Cholecystostomy drains were placed in 16 patients treated by General Surgery ‘on call’ (16/213 patients, 7.5%) versus 7 patients treated in centers with a dedicated ACS service line (7/125, 5.6%, *p* = 0.219).

### Critical care, thromboprophylaxis, and stress ulcer prophylaxis

Five deaths were recorded (1.4%)—one in a patient with a diagnosis of pancreatitis, two in patients with cholangitis and two with acute cholecystitis (AAST Grade IV). Just one case of postoperative mortality happened following cholecystectomy for AAST grade IV cholecystitis.

Nineteen patients (5.6%) required ICU admission for organ failure during their hospital stay—13 (68.4%) of them required inotropic support, 9 (47.4%) required ventilatory support and one (5.3%) required hemodialysis; of these, three (15.8%) had multiorgan failure requiring two or more supports. Three patients required re-admission to the ICU following transfer to the ward—all three were previously admitted with multiorgan failure (*p* < 0.001). Median length of ICU stay was 3 days (mean ± SD, range = 5 ± 5 days, 1–20). The mean APACHE II score for patients admitted to ICU was 17 ± 8.

Subcutaneous low molecular weight heparin was prescribed for venous thromboembolism (VTE) prophylaxis in 270 (79.9%) cases, thromboembolic deterrent stockings (TEDS) were prescribed in 244 (72.2%) patients, unfractionated heparin was prescribed in 8 (2.4%) patients, and 60 (17.8%) patients did not receive mechanical, or pharmacological VTE prophylaxis.

Proton pump inhibitors were prescribed for stress ulcer prophylaxis in 235 (69.5%) patients, ranitidine was prescribed in 14 (4.1%) patients and stress ulcer prophylaxis was omitted from the patient care bundle in 89 (26.3%) patients.

Lactated Ringer’s/Hartmann’s solution was the intravenous fluid used in 167 (49.4%) patients, 0.9% normal saline (with or without supplemental electrolytes) was used in 117 (34.6%), 5% dextrose / balanced electrolyte solution (isolyte M) in 52 (15.4%) and colloid solutions were used in two patients (0.6%). Mean cumulative iv fluid prescription in the first 24 h of hospitalization was 1.64 ± 1.1 L, 3.39 ± 2.68 L at 3 days and 4.25 ± 3.83 L at 5 days.

### Microbiology and antimicrobial therapy

Biological specimens (blood or bile) were sent for microbiological culture and analysis in 154 (54.4%) patients. Specimens were not significantly more likely to have been sent with any particular diagnosis (*p* = 0.210). Blood cultures were sent more frequently than bile cultures, presumably reflecting the fact that bile cultures are obtained intraoperatively and just half of the patients came to operation during the study period (Table 5).

The organisms grown on blood or bile culture were, in descending order of incidence: *E. coli* (11 blood, 20 bile), *Klebsiella *spp*.* (2 blood, 4 bile), *Staphylococcus *spp. (3 blood, 3 bile), *Streptococcus *spp*.* (0 blood, 4 bile), *Enterococcus *spp*.* (0 blood, 4 bile), *Pseudomonas *spp*.* (1 blood, 1 bile), other organisms (2 blood, 6 bile). No growth was recorded in 61 (50%) blood and 69 bile cultures. Polymicrobial blood cultures were seen in one patient and polymicrobial bile cultures in 10 patients (Table 5).

Antimicrobial pharmacotherapy was commenced in 307/338 (90.8%) patients. The most frequently prescribed antibiotic was piperacillin/tazobactam (*n* = 133, 39.3% patients), followed by amoxicillin/clavulanate monotherapy in 65 (19.2%) patients, meropenem monotherapy in 23 (6.8%) patients, metronidazole monotherapy in 15 (4.4%) patients, cephalosporin monotherapy in 14 (4.1%) patients, ciprofloxacin monotherapy in 13 (3.8%) patients, and gentamycin monotherapy in 10 (3.0%) patients. Combination therapies were also seen, with amoxicillin/clavulanate and metronidazole combination therapy in 6 (1.8%) patients, ciprofloxacin and metronidazole combination therapy in 11 (3.3%) patients, cephalosporin and metronidazole combination therapy in 11 (3.3%) patients, and amoxicillin/clavulanate and gentamycin combination therapy in 6 (1.8%) patients. Just 31 (9.2%) patients did not receive any antimicrobial therapy during their hospital stay (Table 5).

### Histopathology

Histopathologic analysis of gallbladder specimens received from 169 operations were reported as acute calculous cholecystitis in 73 (43.2%), acute-on-chronic cholecystitis in 50 (29.6%), gangrenous cholecystitis in 39 (23.1%), gallbladder empyema in 5 (2.9%), xanthogranulomatous cholecystitis in two (1.2%). Atypia of the gallbladder mucosa was noted in one (0.6%) case; there were no cases of gallbladder carcinoma (Table 6).

**Table 6:** Histopathology findings in gallbladder resection specimens

Histopathology findings	Total
Acute calculous cholecystitis	73
Gangrenous cholecystitis	39
Acute on chronic cholecystitis	49
Gallbladder empyema	5
Xanthogranulomatous cholecystitis	2
Atypia of bile duct epithelium	1
Total	169

## Discussion

This first ‘snapshot audit’, a nonrandomized consecutive prospective cohort study drawing together 25 centers from 9 countries, demonstrates the power of collaboration within surgical societies, such as ESTES, to better understand the epidemiology and natural history of a common surgical disease, and to highlight different treatment practices. This prospective collaborative observational cohort study has allowed detailed, defined datasets to be accrued in line with a priori analyses stated in prepublication, open access protocols filed with clinical trial repositories, but is subject to the limitations of its design in only accruing data explicitly detailed in the protocol. The current report, while purely descriptive, highlights some important differences in practices for acute cholecystitis that cannot be explained by individual patient characteristics alone. In particular, an unintended limitation of the current study design—a snapshot in time with a short follow-up (i.e., just 60 days)—is that data regarding the frequency of recurrent admission in patients undergoing nonoperative management is not captured; this is a regrettable shortcoming as recidivism is common in acute cholecystitis. Prior work suggests a failure of nonoperative management within 3–6 months at 24–30% [18, 19], although estimates of early failures are lower [20]. Although 60% of centers in this study self-reported a tendency towards interval cholecystectomy for a variety of operational reasons [21], omission of this metric in the present study should not be inferred as advocating a default approach of interval cholecystectomy.

Although highly recommended by several different guidelines and societies, only 50% of patients followed by this study underwent cholecystectomy during the index hospitalization [21]. Furthermore, heterogeneity in obtaining appropriate and timely microbial culture specimens and potential gaps in antimicrobial stewardship are highlighted by the current study. Although the morbidity and mortality revealed in this study is in keeping with the published literature that informs consensus guidelines, such as the Tokyo Guidelines 2018, the timing of operation and its effects on the outcomes should be further prospectively investigated [3, 6].

Another observation from this study was that centers employing an Acute Care Surgery service line (compared to the traditional ‘on call’ model of care provided by an elective General Surgery service) had a decreased time to intervention, that could lead to decreased length of postoperative hospital stay which may accrue cost savings [2, 5]. Although a time-driven activity-based costing (TDABC) analysis [22] would be prohibitively complex in retrospect across so many international domains and models of care provision in this current study, the learnings provided by our snapshot may guide future work into improving time and cost efficiencies in the provision of emergency general surgical care. The total length of hospital stay (calculated for all patients of all ages and including all diagnoses) did not differ between models of care; this was at odds with a significantly-reduced postoperative length of stay for patients operated on in centers with ACS service lines. It must be presumed, however, that the reasons for this are multifactorial, and are also influenced by diagnosis as well as patient-level factors, such as age-adjusted Charlson Comorbidity Index and by individual outliers.

More than 16% of patients underwent open cholecystectomy, either planned, or converted from laparoscopic. Nine bile duct injuries were reported in 169 cholecystectomies (152 index + 17 interval), an incidence of 5.3%. The vast majority (7/9, 78%) were Strasberg Grade A leaks, leaving two serious BDIs (1 grade D and 1 grade E2) incidence of 1.18%. The true denominator of all cholecystectomies performed in these centers is likely much higher as patients with AAST grade I (uncomplicated) cholecystitis, biliary colic, and biliary dyskinesia were excluded from the study; thus, the true incidence in the population is unknown but is almost certainly much closer to those seen in the large administrative databases quoted in the literature.

As the population of Europe ages (it is estimated that some 30% will be over the age of 65 by 2050), it is important that societies such as ESTES explore clear pathways and guidance for the optimum care of the elderly patient requiring emergency surgery [23–25]. Observations from this snapshot audit (treated in detail elsewhere, REF) that morbidity, postoperative length of stay and the requirement for discharge to a rehabilitation facility are more common in our elderly patients will inform future study on frailty, predictors of morbidity, and optimization of this patient population [26, 27].

Choledocholithiasis, a serious sequel of gallstone disease which may be complicated by cholangitis or biliary pancreatitis, was seen frequently in this snapshot audit. Endoscopic duct clearance is the mainstay of treatment, most commonly followed by definitive management of the stone reservoir by cholecystectomy. Although most of patients (89%) followed this sequence, 11% had one-stage approach with simultaneous laparoscopic cholecystectomy and on-table ERCP; there are favorable data emerging which should prompt consideration of this approach where technically and logistically feasible [28–30].

While not an a priori primary or secondary outcome of the study, our data highlights the heterogeneity in the standard prescription of mechanical and pharmacologic thromboprophylaxis and PPI-based stress ulcer prophylaxis in emergency surgical patients, as mandated in NSQIP care bundles; indeed over 20% of patients in the study did not receive these treatments. This is an important aspect of bundled care that needs further investigation to inform educational efforts [31–33].

At the conception of this collaborative ESTES snapshot audit, we hypothesized that while regional and patient heterogeneity may account for some of the variability that could be expected in different clinical practices to treat these conditions, other causes, such as unit policies and individual surgeon preference might also influence the treatment decisions [34, 35]. This inaugural ESTES snapshot audit has been successful in addressing these questions, and proving the feasibility of international collaborative efforts, and we propose it as a blueprint for future investigation. Further work outside of the scope of the current report is required to delve into the nuances of individual unit and surgeon practices, and the granular ‘real world’ data from snapshot audits, such as this is hypothesis generating in informing prospective case–control studies or even randomized control trials.
