# Correction to: Patterns of prevalence and contemporary clinical management strategies in complicated acute biliary calculous disease: an ESTES ‘snapshot audit’ of practice

**DOI:** 10.1007/s00068-021-01650-y

**Published:** 2021-04-20

**Authors:** Gary Alan Bass, Amy Gillis, Yang Cao, Shahin Mohseni, for the European Society for Trauma, Emergency Surgery (ESTES) Cohort Studies Group, A. Shamiyeh, L. Rosetti, G. Klimbacher, B. Klugsberger, P. Healy, C. Moriarty, C. Power, N. Knightly, A. D. K. Hill, D. C. Winter, M. E. Kelly, B. E. Creavin, É. J. Ryan, C. C. Duffy, M. Sugrue, M. H. Moore, L. Flanagan, J. Ryan, C. Keady, B. Fahey, K. L. McKevitt, K. Barry, K. C. Conlon, K. Mentor, A. Kazemi-Nava, B. J., P. F. Ridgway, D. O. Kavanagh, M. Whelan, M. Donnelly, C. McCarrick, U. Muhammad, T. M. Connelly, P. C. Neary, S. Magalina, V. Cozza, A. LaGreca, D. Gui, A. Malagnino, M. Zago, M. Montuori, A. Biloslavo, N. Samardzic, S. Fracon, D. Cosola, N. de Manzini, U. Fernandes, P. Avelar, R. Marques, A. S. Esteves, A. Marçal, C. Gomes, D. Machado, T. Teles, S. Neves, M. Semiao, R. Cunha, J. Pereira, J. Constantino, M. Sá, C. Casimiro, L. Ionescu, R. Livadariu, L. Stirbu, R. Danila, D. Timofte, B. Astefaniei, A. Landaluce Olavarria, B. Estraviz Mateos, J. Gonzalez Taranco, D. Gomez, J. Barrutia, J. Zeballos, D. Morales Garcia, A. Lozano Najera, E. Gonzalez Tolaretxipi, L. Tallon-Aguilar, J. Pintor-Tortolero, A. Sanchez-Arteaga, V. Duran-Muñóz Cruzado, V. Camacho-Marente, J. Tinoco-Gonzalez, A. Älverdal, S. Redeen, S. Mohseni, A. Mohammad, R. Ahl, M. Wikström, S. Marinos, N. Warner, R. Patel, T. Magro, R. Sunthareswaran, A. Mihailescu, G. Pokusewski, A. L. Bubuianu, C. Dimitriu, M. Paraoan, A. Desai, K. Jones, M. Mlotshwa, K. Ross, S. Lambracos, Y. Tryliskyy, D. C. Cullinane

**Affiliations:** 1 Emergency Surgery Committee, European Society for Trauma and Emergency Surgery (ESTES), Pölten, Austria; 2 Department of Surgery, Tallaght University Hospital, Dublin 24, Ireland; 3 Department of Surgery, Örebro University School of Medical Sciences, Örebro, Sweden; 4 Department of Traumatology, Surgical Critical Care and Emergency Surgery, University of Pennsylvania, Philadelphia, USA; 5 Department of Clinical Epidemiology and Biostatistics, School of Medical Sciences, Örebro University, Örebro, Sweden

## Correction to: European Journal of Trauma and Emergency Surgery 10.1007/s00068-020-01433-x

The original version of this article unfortunately contained a copy-editing mistake. The collaborators of the “European Society for Trauma, Emergency Surgery (ESTES) Cohort Studies Group” were tagged incorrectly in the metadata.

The original article has been corrected.

